# *Rice black-streaked dwarf virus *P6 self-interacts to form punctate, viroplasm-like structures in the cytoplasm and recruits viroplasm-associated protein P9-1

**DOI:** 10.1186/1743-422X-8-24

**Published:** 2011-01-18

**Authors:** Qian Wang, Tao Tao, Yanjing Zhang, Wenqi Wu, Dawei Li, Jialin Yu, Chenggui Han

**Affiliations:** 1 State Key Laboratory for Agro-biotechnology and Ministry of Agriculture Key Laboratory for Plant Pathology, China Agricultural University, Beijing 100193, P. R. China

## Abstract

**Background:**

*Rice black-streaked dwarf virus *(RBSDV), a member of the genus *Fijivirus *within the family *Reoviridae*, can infect several graminaceous plant species including rice, maize and wheat, and is transmitted by planthoppers. Although several RBSDV proteins have been studied in detail, functions of the nonstructural protein P6 are still largely unknown.

**Results:**

In the current study, we employed yeast two-hybrid assays, bimolecular fluorescence complementation and subcellular localization experiments to show that P6 can self-interact to form punctate, cytoplasmic viroplasm-like structures (VLS) when expressed alone in plant cells. The region from residues 395 to 659 is necessary for P6 self-interaction, whereas two polypeptides (residues 580-620 and 615-655) are involved in the subcellular localization of P6. Furthermore, P6 strongly interacts with the viroplasm-associated protein P9-1 and recruits P9-1 to localize in VLS. The P6 395-659 region is also important for the P6-P9-1 interaction, and deleting any region of P9-1 abolishes this heterologous interaction.

**Conclusions:**

RBSDV P6 protein has an intrinsic ability to self-interact and forms VLS without other RBSDV proteins or RNAs. P6 recruits P9-1 to VLS by direct protein-protein interaction. This is the first report on the functionality of RBSDV P6 protein. P6 may be involved in the process of viroplasm nucleation and virus morphogenesis.

## Background

*Rice black-streaked dwarf virus *(RBSDV), an important pathogen that belongs to the genus *Fijivirus *in the family *Reoviridae*, causes rice black-streaked dwarf and maize rough dwarf diseases, which lead to severe yield losses of crops in southeast Asian countries [1-4]. The virus is transmitted to graminaceous plant species via the planthopper *Laodelphax striatellus *in a persistent, circulative manner [4-6]. Typical symptoms caused by RBSDV include stunting, darkening of leaves and white tumours or black-streaked swellings along the veins on the back of the leaves, leaf blades and sheaths. Microscopy of ultrathin sections has shown that the virions are restricted to the phloem tissues in infected plants and that viroplasms, virus crystals and tubular structures are abundantly synthesized in both infected plants and insect cells [1,4,7,8].

The RBSDV virion is an icosahedral, double-layered particle with a diameter of 75-80 nm and consists of ten genomic dsRNA segments [9-12]. Protein sequence analysis suggested that S1 encodes a putative 168.8-kDa RNA-dependent RNA polymerase. S2 and S4 encode a core protein and an outer-shell B-spike protein, respectively [8,11,12]. The protein encoded by S3 is assumed to have some guanylyltransferase activity [13]. Proteins translated from S8 and S10 are the components of the major capsid and outer capsid, respectively [8,14,15]. Both S7 and S9 encode nonstructural proteins. S7 ORF1 P7-1 and S9 ORF1 P9-1 are components of the tubular structures and viroplasm produced in infected cells, respectively [8]. Recent studies have demonstrated that P9-1, an *α*-helical protein with a molecular mass of 40 kDa, self-interacts to form dimers, and it is proposed to be the minimal viral component required for viroplasm formation [16]. P6 is a large nonstructural protein containing 792 amino acids with a molecular mass of 89.6 kDa that is translated from S6, which is 2645 bp in length and contains a single long ORF. It is synthesized abundantly in RBSDV-infected plants and viruliferous planthoppers [17]. However, further characterization and elucidation of the functions of P6 have not yet been reported.

In this study, we investigated the homologous interaction P6-P6 using a yeast two-hybrid (YTH) assay and bimolecular fluorescence complementation assay (BiFC) and determined the subcellular localization of P6 and P6 derivatives using two different fluorescent markers. P6 self-interacts and forms large discrete viroplasm-like structures (VLS) in plant cytoplasm. The minimal region of P6 necessary for P6 self-interaction *in vivo *is composed of amino acids residing between positions 395 and 659. The exact residues in this region that greatly affect the subcellular distribution of P6 were also determined. Furthermore, a strong interaction between P6 and the viroplasm-associated protein P9-1 was apparent from YTH analyses and co-expression experiments. These results might provide deeper understanding of the process of viroplasm formation of RBSDV.

## Results

### P6 forms punctate, cytoplasmic viroplasm-like structures *in vivo *and self-interacts in YTH system

**Figure 1:**
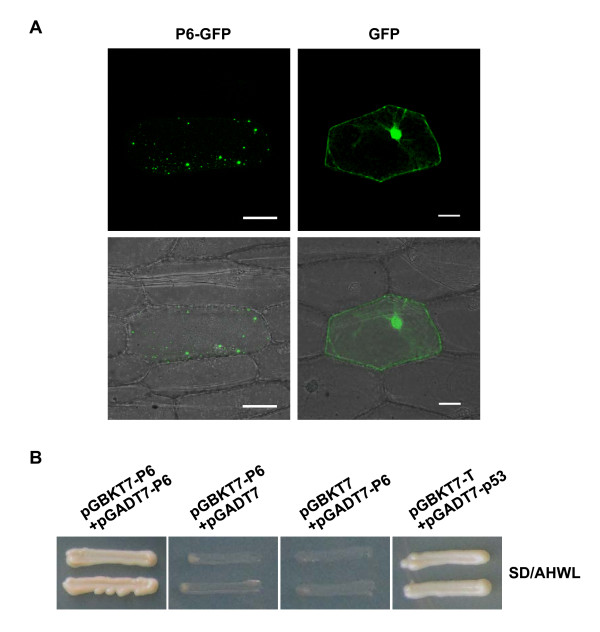
**P6 forms punctate, cytoplasmic VLS in the onion epidermal cells and self-interacts in YTH system**. (A) Subcellular localization of RBSDV P6 fused to GFP and free GFP in onion epidermal cells. Punctata VLS of different sizes were prevalently formed in the onion cells expressing P6-GFP, while diffuse GFP fluorescence was observed in the nucleus and cytoplasm of the cells expressing free GFP. The results were observed 16-24 h after particle bombardment. Bars, 50 μm. (B) Yeast colonies containing pGBKT7-P6/pGADT7-P6 grew well on the selective medium as did yeast colonies containing pGBKT7-T/pGADT7-p53, which was used as the positive control, whereas yeast transformed with pGBKT7-P6/pGADT7 or pGBKT7/pGADT7-P6 used as negative controls were unable to grow.

To determine the subcellular localization of P6, the plasmid expressing P6 fused with green fluorescent protein (GFP) at its C terminus (P6-GFP) was introduced into onion epidermal cells by particle bombardment. Confocal fluorescence microscopy analysis indicated that abundant, punctate viroplasm-like fluorescent foci were observed in the cytoplasm of the onion cells. The bright discrete foci were of different sizes and scattered in the cytoplasm. No apparent fluorescence was visualized in the nuclei. As a negative control, free GFP resulted in a diffuse pattern of fluorescence that was both nuclear and cytoplasmic, which indicated that the moiety GFP does not affect the localization of P6-GFP (Figure 1A). Identical results were observed when the proteins were expressed in the protoplasts of *Nicotiana. benthamiana *(Additional file 1, Figure S1). This demonstrated that P6 tends to aggregate to form structures that resemble the matrix of the viroplasm when expressed in the absence of other RBSDV proteins, and led us to speculate that P6 might self-associate and be involved in the formation of the viroplasm.

Subsequently, a YTH assay was performed to find out whether P6 had an intrinsic ability to self-interact *in vivo*. Combinations of plasmids expressing bait protein BD-P6 and prey protein AD-P6 were transformed into Y187 and AH109 strains, respectively. Making sure there was no transcriptional activation or toxicity of BD-P6 for yeast strains, western blot analysis was carried out to verify that both BD-P6 and AD-P6 were expressed in the yeast (data not shown). Cotransformation and yeast mating assays showed that independent yeast colonies containing pGADT7-P6 and pGBKT7-P6 grew well and turned blue in the *β*-galactosidase colony-lift filter assay (data not shown), indicating that there were strong interactions between P6 molecules. In contrast, no growth was observed for the negative controls (Figure 1B). This suggested that P6 has an inherent ability to self-interact and is able to form VLS when expressed alone in plant cells.

### YTH assays indicate the centrally located region spanning residues 395 to 659 is necessary for P6 self-interaction

As there was not much information available from the literature about P6, protein sequence analysis was performed. BLAST searches indicated that the region approximately inclusive of residues 400 to 675 exhibited limited conservation of amino-acid sequence with the ATPase domain of structural maintenance of chromosomes proteins (SMCs), which play an essential role in chromosome segregation, condensation and organization [18].

**Figure 2:**
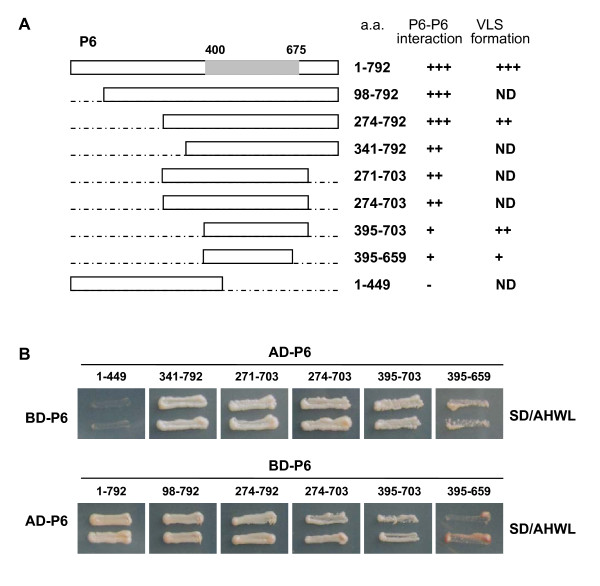
**Mapping of the P6 region involved in P6 self-interaction**. (A) Schematic representation of P6 and P6 truncations in the study. The full-length P6 (spanning residues 1 to 792) and P6 truncations are indicated by open bars. The P6 domain (approximately from position 400 to 675) homologous to SMC ATPase is indicated by the gray bar and the deleted regions by the dashed lines. The numbers denote P6 amino acid positions. The ability of P6 truncations to interact with intact P6 in YTH assays is indicated in the middle (+, positive; -, negative). The VLS-forming abilities of the different P6 derivatives are shown on the right (+ + +, abundant and large VLS; + +, moderate in size and number; +, few in number; -, negative with diffuse distribution; ND, not determined). (B) Homologous interaction between intact P6 and P6 deletions in YTH assays. All truncations harbouring this region were able to interact with intact P6. As their N and C termini approached this region, the interaction ability was decreasing.

In order to determine the region necessary for P6-P6 self-interaction, we sequentially constructed a collection of truncation derivatives that express BD-P6^98-792^, BD-P6^274-792^, BD-P6^274-703^, BD-P6^395-703^, BD-P6^395-659^, AD-P6^1-449^, AD-P6^341-792^, AD-P6^271-703^, AD-P6^274-703^, AD-P6^395-703 ^and AD-P6^395-659^, based on the protein sequence analysis results. Homologous binding capabilities between P6 and these deletions were investigated via the YTH assay. Schematic representation of the different P6 truncations is shown in Figure 2A.

The YTH analysis indicated that a centrally located domain between positions 395 and 659 was required for P6-P6 interaction. All truncations harbouring this region were able to interact with intact P6. However, as their N and C termini approached this region, the abilities of the P6 mutants to associate with intact P6 decreased. Varying interaction abilities were indicated by the rates of yeast growth on the selective medium. When the deletion comprised exactly the region from positions 395 to 659, the interaction with P6 was very weak, and the colonies transformed with pGADT7-P6^395-659^/pGBKT7-P6 or pGADT7-P6/pGBKT7-P6^395-659 ^showed obvious growth inhibition and the streaks turned dark red. Mutant P6^1-449^, in which most of the central and C-terminal region was deleted, showed complete inability to interact with P6 (Figure 2B). Binding capabilities between these deletions were also investigated, and the results demonstrated that, even when both the N and C termini were absent, the deletions had some ability to associate with each other (data not shown). The results suggested that the region from residues 395 to 659 is necessary to sustain the P6 self-interaction and that further truncation might abolish this interaction.

### Transient expression experiments of P6 derivatives indicate residues 395 to 659 are important for P6 self-interaction

Recombinant plasmids that can express P6^274-792^, P6^395-703 ^and P6^395-659^, fused in-frame to the N terminus of GFP (P6^mutant^-GFP) or the C terminus of DsRed2 (DsRed-P6^mutant^), were constructed and their subcellular localization was determined. Plasmids expressing P6^mutant^-GFP were delivered into onion epidermal cells via biolistic bombardment, whereas those expressing DsRed-P6^mutant ^were introduced into epidermal cells of *N. benthamiana *leaves by agroinfiltration assay [19].

**Figure 3:**
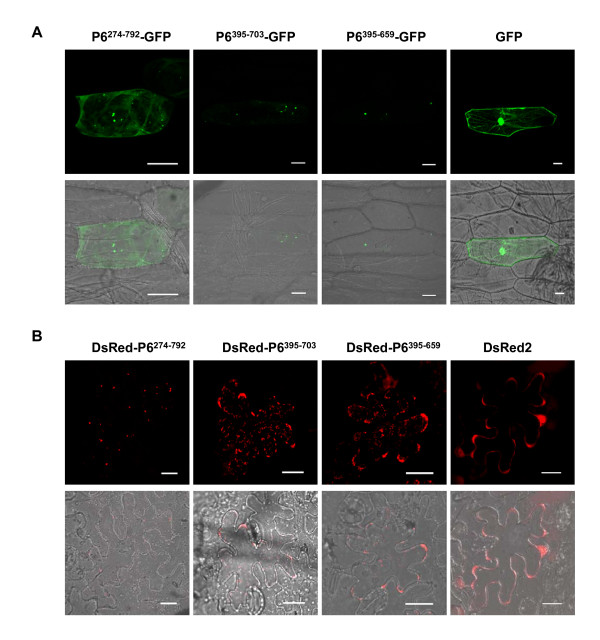
**Distribution of P6 truncated versions *in planta***. (A) Subcellular localization of P6 truncations fused with GFP and free GFP in onion epidermal cells. GFP was excited at 488 nm and emission was measured at 550-590 nm. Bars, 50 μm. (B) Subcellular localization of P6 truncations fused with DsRed2 and free DsRed2 in the epidermal cells of *N. benthamiana *leaves. DsRed2 was excited at 543 nm and emission was measured at 570-600 nm. Bars, 20 μm. The fluorescence and merged images are depicted in the upper and lower panels, respectively.

Biolistic bombardment experiments indicated that P6^274-792^-GFP mostly formed large bright discrete foci in the cytoplasm of onion cells, but low levels of diffuse cytoplasmic fluorescence were also observed. P6^395-703^-GFP expression resulted in the formation of irregular aggregate-like structures, and minor levels of diffuse GFP signals were also observed at the peripheries of the nuclei, P6^395-659^-GFP resulted in very few (generally less than five) discrete and bright foci in the cytoplasm (Figure 3A). Similar results were obtained when these mutants fused with DsRed2 were expressed in the epidermal cells of tobacco leaves (Figure 3B) or tobacco protoplasts (Additional file 2, Figure S2). Numerous dispersed punctate VLS were detected in the tobacco cells expressing DsRed-P6^274-792^, and the expression of DsRed-P6^395-703 ^and DsRed-P6^395-659 ^resulted in amounts of irregular aggregate-like foci. Weak and uniform red fluorescence signals were present in the cells expressing free DsRed2.

Generally, the fluorescence distribution patterns of the three mutants (P6^274-792^, P6^395-703 ^and P6^395-659^) indicated that the 395-659 region is important for P6 localization and that self-assembly is possible outside of the P6 native environment. The results also suggested that residues on both sides of the 395-659 region might be engaged in the process, based on the numbers and the size of the fluorescent foci.

### Bimolecular fluorescence complementation assay confirms that P6 molecules self-interact *in planta*

**Figure 4:**
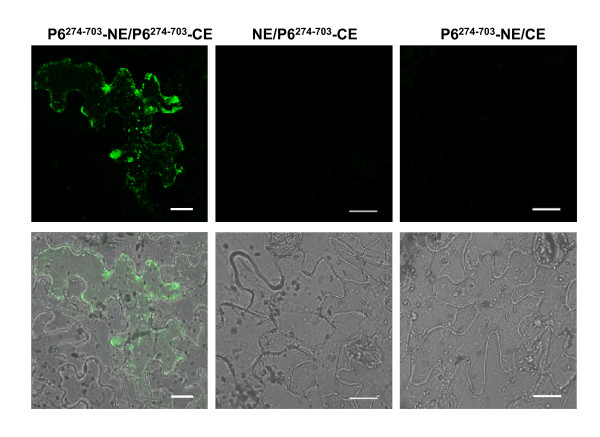
**BiFC visualization of P6^274-703 ^interaction in agrobacterium-infiltrated *N. benthamiana *leaves**. Co-expression of P6^274-703^-YN and P6^274-703^-YC induced strong recovered YFP signals in the cytoplasm, and no YFP signals were detected for the negative controls following the co-expression of P6^274-703^-YN/YC or P6^274-703^-YC/YN. YFP was excited at 488 nm and emission was measured at 550-590 nm. The fluorescent and bright field images are depicted in the upper and lower panels, respectively. Bars, 20 μm.

In order to determine whether P6 molecules self-interact *in planta*, bimolecular fluorescence complementation assays were carried out (Figure 4). One pair of combinations that can express P6^274-703 ^fused either to YN or YC was constructed and then delivered into *N. benthamiana *leaves via agroinfiltration. As expected, co-expression of P6^274-703^-YN and P6^274-703^-YC induced strong recovered YFP signals, which formed numerous tiny fluorescent sites or irregular aggregate-like structures in the cytoplasm. No YFP signals were detected for the negative controls following the co-expression of P6^274-703^-YN/YC or P6^274-703^-YC/YN. The BiFC assay provided strong evidence that the truncated mutant P6^274-703 ^participates in self-interaction so that recovered YFP signals are detected easily in the tobacco cells. From these results, we can confirm that P6 molecules have the ability to self-interact *in planta*.

### Polypeptides consisting of residues 580 to 620 and 615 to 655 are involved in VLS formation

**Figure 5:**
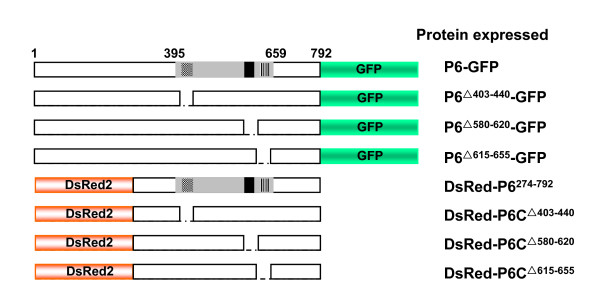
**Schematic representation of P6 deleted versions fused with GFP or DsRed2**. The full-length P6 and its deleted versions are indicated by open bars and the deleted regions by dashed lines. The numbers denote P6 amino acid positions. P6 395-659 fragment is indicated by the gray bar and the three predicted motifs designated pumilio RNA-binding repeat profile, sialic-acid binding micronemal adhesive repeat and intra-flagellar transport protein 57 are indicated by the checkered, black, and hatched boxes, respectively. GFP and DsRed2 are indicated by the green and red bars, respectively.

In light of the results above, it is evident that P6^395-659^, which only constitutes one-third of the entire P6 protein, is essential to P6 self-interaction. It is possible that some specific elements in this fragment are responsible for the VLS formation. A P6 motif prediction using My-Hits scan http://www.expasy.cn showed that three putative motifs might have relatedness to this interacting region. These three putative motifs are designated pumilio RNA-binding repeat profile, sialic-acid binding micronemal adhesive repeat and intra-flagellar transport protein 57, and they correspond to P6 residues 401-439, 584-608 and 624-654, respectively. In addition, the secondary structure prediction demonstrated that a putative coiled-coil motif might reside in the region from residues 550 to 640. To determine which motifs might be involved in VLS formation, corresponding derivatives that express P6^△403-440^-GFP, P6^△580-620^-GFP, P6^△615-655^-GFP, DsRed-P6C^△403-440^, DsRed-P6C^△580-620 ^and DsRed-P6C^△615-655 ^were constructed and their subcellular localization was investigated. It is noteworthy that we did create several plasmids aiming to express intact P6 fused with DsRed2 but failed to detect the fused protein for unknown reasons. Previous results showed that DsRed-P6^274-792 ^was sufficient to induce inclusion bodies, so we created the corresponding mutants (DsRed-P6C^△403-440^, DsRed-P6C^△580-620 ^and DsRed-P6C^△615-655^) based on this abridged construction. Schematic representation of the different P6 deletion derivatives is shown in Figure 5. As described earlier, plasmids expressing P6^mutant^-GFP were bombarded into onion cells, while those expressing DsRed-P6^mutant ^were introduced into tobacco leaves by agroinfiltration assay.

**Figure 6:**
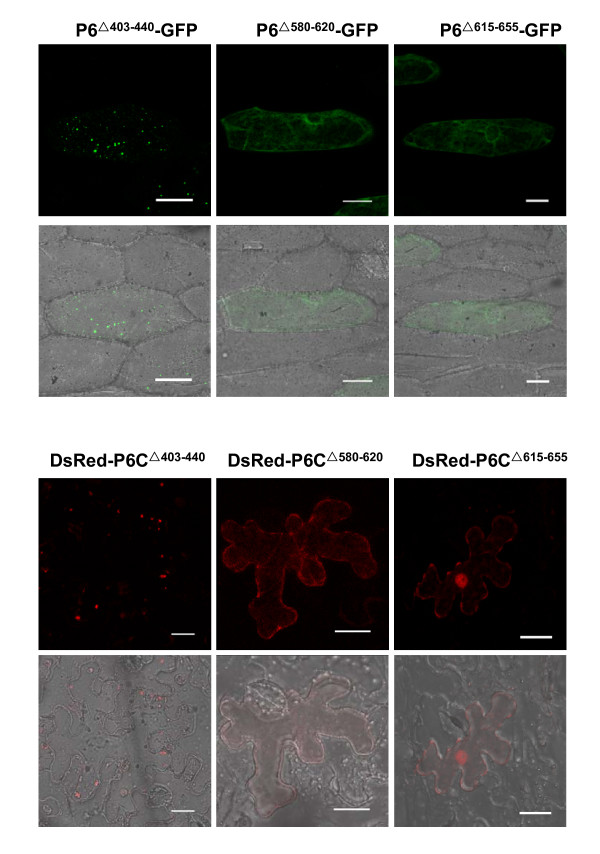
**Transient expression results of P6 deleted derivatives**. The upper two panels indicate the distribution of P6 deletions fused with GFP expressed in the onion epidermal cells, showing that P6^△580-620^-GFP and P6^△615-655^-GFP have a diffuse fluorescence pattern while P6^△403-440^-GFP forms numerous VLS. GFP was detected with excitation at 488 nm and emission capture at 550-590 nm. Bars, 20 μm. The lower two panels indicate the distribution of DsRed2-fused P6 deletions expressed in the epidermal cells of *N. benthamiana *leaves. Similarly, Both DsRed-P6C^△580-620 ^and DsRed-P6C^△615-655 ^show a diffuse and weak red fluorescence distribution whereas DsRed-P6C^△403-440 ^forms VLS. Red fluorescence was detected with excitation at 543 nm and emission capture at 570-600 nm. Bars, 50 μm.

Confocal fluorescence microscopy showed that P6^△403-440^-GFP accumulated to form numerous punctate bright foci in the cytoplasm, indistinguishable from those induced by P6-GFP. In contrast, P6^△580-620^-GFP and P6^△615-655^-GFP distributed throughout the cytoplasm displaying a weaker fluorescence pattern, compared to free GFP, and the fluorescence signals were always visualized at the periphery of the nuclei. Similar results were obtained when P6 mutants were fused with DsRed2. Numerous dispersed punctate aggregates were detected in the tobacco cells expressing DsRed-P6C^△403-440^, whereas weak and uniform DsRed2 signals were present in the cells expressing either DsRed-P6C^△580-620 ^or DsRed-P6C^△615-655^. The results are shown in Figure 6.

To sum up, two polypeptide chains, comprising residues 580 to 620 and 615 to 655, are implicated in VLS formation, and loss of them alters the subcellular localization of P6.

### YTH assays demonstrate P6 interacts with P9-1

**Figure 7:**
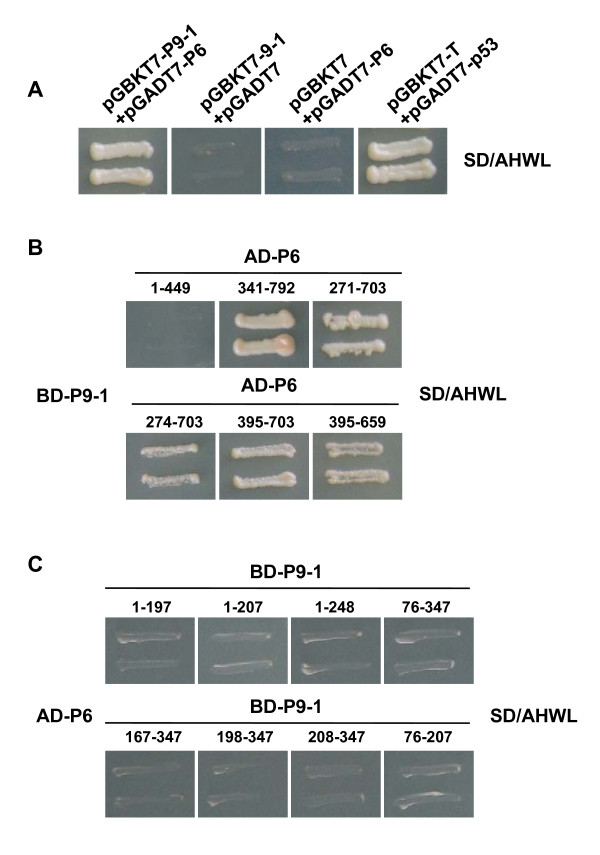
**Investigation of P6-P9-1 interaction in YTH system**. (A) Yeast colonies containing pGBKT7-P9-1/pGADT7-P6 grew well on the selective medium, whereas the yeast transformed with pGBKT7-P9-1 and pGADT7, used as a negative control, was unable to grow. (B) Yeast colonies expressing BD-P9-1 with AD-P6^341-792^, AD-P6^274-703^, AD-P6^271-703^, AD-P6^395-703^, or AD-P6^395-659 ^grew well on the selective medium, but those expressing BD-P9-1 with AD-P6^1-449 ^did not. The numbers denote P6 amino acid positions. (C) Yeast colonies expressing AD-P6 with any of the P9-1 mutants fused with BD domain showed no growth on the selective medium. The numbers denote P9-1 amino acid positions.

Immunoelectron microscopy revealed that antibodies against P9-1 reacted with viroplasm in infected cells [8]. Based on our findings above, P6 likely participates in viroplasm formation. This prompted us to further explore the relationship between P6 and P9-1 via a YTH assay. A plasmid that can express BD-P9-1 was constructed and transformed into Y187 strain. Interestingly, the results showed that there is an intimate association between P9-1 and P6 (Figure 7A). Yeast colonies containing both pGBKT7-P9-1 and pGADT7-P6 grew well on the selective medium, whereas yeast transformed with pGBKT7-P9-1 and pGADT7, which was used as a negative control, was unable to grow. This result indicated that P6 interacts with P9-1 *in vivo*.

### P9-1 cannot form inclusion-like structures when expressed alone

**Figure 8:**
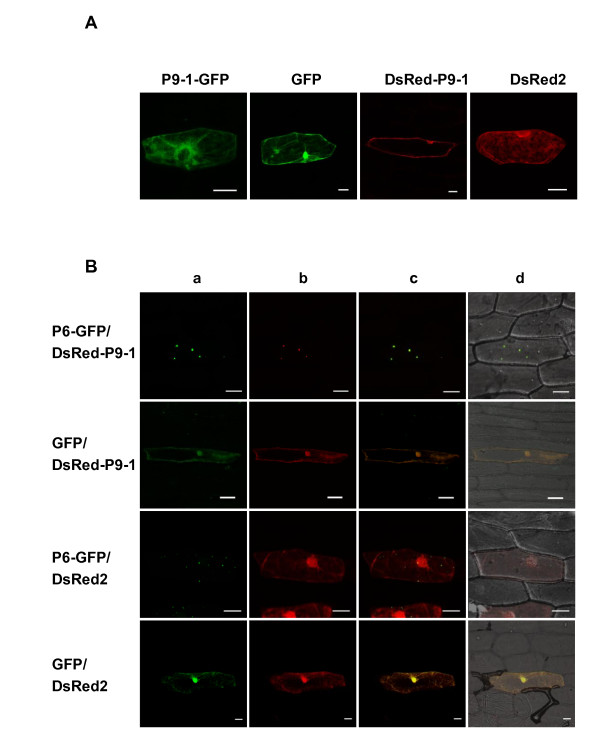
**P6 is able to recruit P9-1 to VLS in onion epidermal cells**. (A) Subcellular localization of P9-1 fused with GFP or DsRed2. P9-1-GFP and DsRed-P9-1 were distributed diffusely in the onion cells and were unable to form inclusion bodies. (B) Co-expression of P6-GFP and DsRed-P9-1 in onion epidermal cells. Detection of green (lane a) and red (lane b) fluorescence was achieved with excitation at 488 nm and 543 nm, respectively; co-localization of green and red fluorescence is indicated in yellow (lane c); superposition of the green and red fluorescence images as well as the bright field image is shown on the right (lane d). The co-expression results indicate that P6 was able to relocate the distribution of P9-1, that both proteins were present exclusively in the discrete and punctate foci, and that expression of DsRed2 or GFP had no aberrant effects on DsRed-P9-1 or P6-GFP distribution. Bars, 50 μm.

Two plasmids that express P9-1-GFP and DsRed-P9-1 were constructed and bombarded into onion epidermal cells to determine P9-1 subcellular localization. Fluorescence microscopy indicated that both P9-1-GFP and DsRed-P9-1 resulted in a pattern of diffuse and uniform fluorescence distribution in the cytoplasm and nuclei of onion cells, which was a little weaker than that of free GFP or DsRed2 controls (Figure 8A). Our results are inconsistent with the conclusion of Zhang et al that P9-1 alone aggregates to form inclusion bodies [16]. The same results were obtained when the plasmids were delivered into tobacco protoplasts via polyethylene glycol (PEG) transfection method or introduced into epidermal cells of tobacco leaves by agroinfiltration assay (Additional file 3, Figure S3). Therefore, we consider that P9-1 has a widespread distribution but no ability to aggregate in the cytoplasm when expressed in plant cells on its own.

### Colocalization experiments indicate P6 relocalizes the distribution of P9-1 and recruits P9-1 to VLS

Co-expression experiments were developed to investigate potential P6-P9-1 interactions (Figure 8B). We introduced two plasmids expressing P6-GFP and DsRed-P9-1 into onion cells by cobombardment. Contrary to the case when DsRed-P9-1 was expressed alone, when P6-GFP and DsRed-P9-1 were co-expressed, a striking relocalization of red fluorescence emerged. DsRed-P9-1 displayed a nearly complete coincidence with the intracellular distribution of P6-GFP. The two proteins were colocalized and exclusively presented in discrete punctate VLS, identical to those formed by P6-GFP alone, and no diffuse green or red fluorescent signals were observed in the cytoplasm or the nuclei. Control combinations were also investigated to rule out the possibility that GFP or DsRed2 expression might have some aberrant effects on the DsRed-P9-1 or P6-GFP distribution. The colocalization of P6-GFP and DsRed-P9-1 confirmed that P6 has a dramatic effect on the distribution of P9-1 and that it is caused by the direct association between these two proteins.

### YTH assays confirm residues 395 to 659 of P6 are necessary for P6-P9-1 heterologous interaction

Further YTH analyses were performed to examine the regions of P6 crucial for P6-P9-1 heterologous interaction. P6 AD-fused deletions, including AD-P6^1-449^, AD-P6^341-792^, AD-P6^274-703^, AD-P6^271-703^, AD-P6^395-703 ^and AD-P6^395-659^, were tested and all P6 deletions except AD-P6^1-449 ^were able to interact with P9-1. Transformants expressing BD-P9-1 and AD-P6^1-449 ^showed no growth on the selective medium, whereas those containing other combinations grew well (Figure 7B). The results indicated that the region located between amino acids 395 and 659 is indispensable for P6-P9-1 interaction.

### YTH assays indicate deletion mutants of P9-1 do not interact with P6

We also investigated P9-1 regions crucial for P6-P9-1 interaction. A dozen P9-1 BD-fused deletions that express fusions BD-P9-1^1-197^, BD-P9-1^1-207^, BD-P9-1^1-248^, BD-P9-1^76-347^, BD-P9-1^167-347^, BD-P9-1^198-347^, BD-P9-1^208-347 ^and BD-P9-1^76-207 ^were constructed. YTH results indicated that all deletions completely lost the ability to interact with P6 (Figure 7C). It is supposed that minor changes in the protein sequence might affect the properties and protein structure of P9-1 and thereby abrogate P6-P9-1 interaction.

## Discussion

Compared to animal reoviruses, most events in the Fijivirus life cycle, such as virus entry, replication, and packaging and particle assembly and systemic movement, are poorly understood, as are the functions of proteins encoded by the viral genome. In this study, we investigated the uncharacterized protein P6 of RBSDV, a member of the *Fijivirus *genus, by employing the related experiments in protein-protein interactions.

YTH analysis and/or subcellular localization experiments showed that P6 interact (Figure 2B) and establish punctate VLS when solely expressed in plant cells (Figure 1; Additional file 1, Figure S1), and BiFC assays also indicated that the truncated version P6^274-703 ^(equivalent to one-third of the whole P6 protein) is able to interact intimately to form aggregate-like structures (Figure 4). These results, which clearly demonstrated that P6 has a strong ability to self-assemble, prompted us to question whether P6 is capable of forming multimeric structures. Multimerization of viral proteins always plays an essential role in the virus cycle [20-22]. In *Reoviridae*, the viroplasm determinants, such as NSP2 and NSP5 of rotaviruses, μNS and σNS of orthoreoviruses, NS2 of orbiviruses, and Pns12 of rice dwarf virus, all share this characteristic to assemble into higher-order complexes to recruit other viral proteins or RNAs [23-29]. The self-interaction of RBSDV P6 might be prerequisite for its multimerization and subsequently for its biological functions.

The coiled-coil region might be involved in P6-P6 interactions. Coiled-coil motifs are increasingly recognized as key determinants in both intra- and inter-molecular interactions. In our experiments, the P6 region spanning residues 365 to 659, which is predicted to harbour a coiled-coil structure and show some sequence homology with the ATPase domain of SMCs, is crucial for VLS formation. Deleting two peptide chains (aa 580-620 and aa 624-654) abolishes VLS formation (Figure 6), which suggests that loss of this region might have a pronounced effect in altering the context of the whole protein and perturb the correct folding of the coiled-coil domain and thereby inhibit molecular interactions. On the basis of the different rates of yeast growth in the YTH assay and the different numbers of fluorescent foci formed in transient expression experiments, we conclude that, whereas the central region spanning residues 365 to 659 is identified as important for P6-P6 or P6-P9-1 interactions, the amino acid sequences near to this region might also affect these interactions by changing the stability of the newly-built protein complexes.

A strong interaction between P6 and P9-1 was detected in our experiments. The two proteins are both expressed at high levels in infected plants and viruliferous insects, as detected by using antibodies against them [8,17]. Previous experiments indicated that the viroplasm matrix was densely and evenly immunolabelled with antibodies against P9-1 [8]. Although corresponding electron microscopy results have not been obtained for P6, the ability of P6 to form VLS and the heterologous interaction between P6 and P9-1, as well as the localization of P9-1 in hosts, hint that P6 might associate with viroplasm and play a role in the viroplasm nucleation. It is noteworthy that orthoreovirus μNS, which plays an essential role in the process of viroplasm formation, is able to assemble into globular VLS when expressed alone and recruit another viroplasm-associated protein σNS to the VLS [24,30,31]. This is quite similar to our results.

Despite the lack of detectable protein sequence homology with animal reovirus proteins, P6 possesses some common features with their viroplasm determinants. Being expressed at high level in hosts, possessing the ability to form VLS and recruiting the viroplasm-associated protein P9-1, P6 protein prediction showed that the P6 fragment located between amino acids 400 and 675 has a low homology with the SMC ATPase domain, whereas the region from positions 404 to 439 is likely to be a pumilio RNA-binding repeat profile, which indicates that P6 might be involved in ATP hydrolysis and binding of RNA. Generally, viroplasm determinants are often inferred to possess NTP-hydrolysis and RNA-binding activities to assist in the process of RNA replication, especially in *Reoviridae *[32,33]. It is necessary to do further work to elucidate the biochemical and biophysical properties of P6 and to determine whether P6 functions according to a mechanism that is similar to other viroplasm determinants of reoviruses in the process of viroplasm formation.

RBSDV P9-1 was previously reported to form inclusion bodies when tagged with GFP at its C-terminus and expressed in *Arabidopsis *protoplasts [16], which is contrary to our findings. We investigated the P9-1 distribution by two different transient expression strategies. Whenever the protein is fused with GFP at its C terminus or DsRed2 at its N terminus and expressed in onion (Figure 8A) or tobacco epidermal cells or tobacco protoplasts (Additional file 3, Figure S3), P9-1 has a diffuse distribution pattern in the cytoplasm and cannot form aggregates. Consistent with the conclusion reached by Zhang et al [16], we confirmed that P9-1 self-interacts in the YTH system and forms stable dimers *in vitro *(data not shown). P9-1 itself might not be the nucleating factor in plant cells for it is located in the VLS only when coexpressed with P6.

Being highly homologous to RBSDV P9-1 (64.5% identity) [34], *Mal de Rio Cuarto virus *(MRCV) P9-1, which was detected in viroplams in both infected plants and planthoppers [35], was found to be sufficient for the formation of viral inclusion body (VIB)-like structures when expressed in *Spodoptera frugiperda *Sf9 cells [36]. There might be distinct mechanisms involved in viroplasm formation in insect and plant hosts. These questions need to be addressed in future work.

## Conclusions

This is the first report on the functionality of RBSDV nonstructural protein P6, which previously was completely uncharacterized. Our results showed RBSDV P6 self-interacts and forms punctate cytoplasmic VLS when expressed alone. Furthermore, P6 strongly interacts with the viroplasm-associated protein P9-1 and recruits P9-1 to localize in VLS. The P6 and P9-1 regions necessary for these homologous or heterologous interactions were also determined, as well as the exact residues essential for P6 VLS formation. Results presented here might provide clues for understanding the viroplasm nucleation of RBSDV and allow us to gain further insight into the relationship between P6 and P9-1 in the virus life cycle.

## Methods

### General

Healthy *N. benthamiana *plants were grown at 23 °C under 1,000 lumens with a 16-hour daylight regimen. *Agrobacterium tumefaciens *strain EHA105 was grown on LB agar containing 50 g/ml rifampin. The yeast strains, *Saccharomyces cerevisiae *AH109 and Y187, and the yeast vectors, pGBKT7 and pGADT7, as well as the positive control plasmids, pGBKT7-T and pGADT7-p53, were used for YTH analyses (Clontech). The binary expression vectors pGDR and pGDp19 used to express *Tomato bushy stunt virus *(TBSV) p19 for suppressing gene silencing were obtained as generous gifts from Professor Andrew O. Jackson of the University of California at Berkeley, USA, while another plasmid, pEGFP (Clontech) which harboured EGFP segment, was kindly provided by Professor Zaifeng Fan, China Agricultural University, PR China. BiFC vectors, pSPYNE-35S and pSPYCE-35S, were kindly provided by Professor Jörg Kudla, Universität Müneter, Germany. Both RBSDV S6 (GenBank: AY144570) and S9 (GenBank: AF536564) full-length cDNA clones were maintained in our lab [11].

### Construction of recombinant plasmids

To generate transient expression vector pGFPI, pBI221 was digested with *Hin*dIII/*Xba*I and *Sac*I/*Eco*RI respectively, and the liberated CaMV 35S promoter and nos terminator were ligated to pUC18-T corresponding clone sites to obtain an intermediate vector. EGFP encoding the autofluorescent protein was amplified using primers EGFP-1/EGFP-2 from plasmid pEGFP (clontech). PCR products were digested with *Kpn*I/*Sac*I, and ligated into the *Kpn*I/*Sac*I-digested pUC18-T intermediate to generate pGFPI. Only the *Xba*I and *Kpn*I can be used to express GFP-tagged protein in this vector. To generate expression recombinants for GFP-tagged P6, full-length of P6 ORF was amplified using a pair of primers PS6-1/PS6-6 (Table 1). PCR products were ligated to pMD19-T to obtain pMD19-T-S6. pMD19-T-S6 was digested with *Bam*HI/*Xho*I, and ligated into *Bam*HI/*Xho*I-digested pSPYNE-35S. The clone was then cut by *Xba*I/*KpnI *and the liberated fragment was ligated into the *Xba*I/*Kpn*I-digested pGFPI to yield pS6GFPI, which can express P6-GFP. P6 deletion and truncation fragments were produced through PCR amplification, using the primers shown in Table 1, and PCR products were ligated to pMD19-T or self-ligated to obtain intermediates selected for further use. The intermediates containing P6 truncation fragments (P6^274-792^, P6^395-703^, P6^395-659^) were digested with *Xba*I/*Kpn*I and the liberated fragments were ligated into *Xba*I/*Kpn*I-digested pGFPI to yield vectors expressing P6 truncations fused with GFP (P6^274-792^-GFP, P6^395-703^-GFP, P6^395-659^-GFP). As in the construction of pS6GFPI, similar strategies were used to obtain three P6 deletion derivatives (expressing P6^△403-440^-GFP, P6^△580-620^-GFP, P6^△615-655^-GFP) and pS9-1GFPI (expressing P9-1-GFP). For expression of the DsRed2-fused proteins, intermediates containing P6 truncation fragments (P6^274-792^, P6^395-703^, P6^395-659^) and those containing P6 deletion fragments (P6C^△403-440^, P6C^△580-620^, P6C^△615-655^) were digested with *Xho*I/*Bam*HI and *Hin*dIII/*Sal*I, respectively, and the liberated fragments were ligated to the corresponding *Xho*I/*Bam*HI- and *Hin*dIII/*Sal*I-treated pGDR to generate vectors expressing P6 truncations and P6 deletions fused with DsRed2. A vector expressing DsRed-P9-1 was constructed similarly to those expressing P6 truncation fused with DsRed2.

**Table 1:** Primers used for PCR amplification.

Primer	Sequence (5′→3′) ^a^	Locations ^b ^and modifications
PGFP-F	ggtacc ATGGGTAAAGGAGAAGAAC	1aa;*Kpn*I

PGFP-R	gagctc TTATTTGTATAGTTCATC	full-length reverse primer with stop codon; *Sac*I

PS6-1-F	CG ggatcc ATGTCTGCCC	1aa; *Bam*HI

PS6-4-F	CTAG ccatgg *GA *ATGTCTGCCCACCTGACCAATTTAG	1aa; *Nco*I

PS6-5-R	CG ggatcc TTACTCAGAGCTTAGTTGCCAGAGG	full-length reverse primer with stop codon; *Bam*HI

PS6-6-R	CCG ctcgag CTCAGAGCTTAGTTGCC	full-length reverse primer without stop codon; *Xho*I

PS6-8-R	CCG ctcgag ATCAGCTACTTCGTCAG	449aa; *Xho*I

PS6-9-F	CG ggatcc *AC *ATGTCTGCCCACCTG	1aa; *Bam*HI

PS6-10-F	CCG ctcgag ccatgg AAGCTTCTGATGTCCAG	274aa; *Xho*I, *Nco*I

PS6-11-F	CCG ctcgag ccatgg ACTTGATTAATCATGCC	395aa; *Xho*I, *Nco*I

PS6-12-R	CG ggatcc ggtacc ATCTCCAAAGTTAGCATCTAC	703aa; *Bam*HI, *Kpn*I

PS6-15-R	CG ggatcc ggtacc CGTTTCATTAGCAGATGTTTTG	659aa; *Bam*HI, *Kpn*I

PS6-16-R	TCC cccggg GAACAGATCGGCATGATTAATC	403aa; *Sma*I

PS6-17-F	TCC cccggg GTGAATGATTTAACTGACGAAG	440aa; *Sma*I

PS6-18-R	CATG gggccc GTCTTTCTCTTTTAGTAAAGAACAG	615aa; *Apa*I

PS6-19-F	CATG gggccc TCTGCTAATGAAACGAATGATG	655aa; *Apa*I

PS6-20-R	CATG gggccc GGCAATCTGTTCTTTAGCTTGTC	580aa; *Apa*I

PS6-21-F	CATG gggccc GAGAACGAAATGTTGAAGGAACAG	620aa; *Apa*I

PS6-24-F	GC tctaga ccatgg ACGTACTCAACCTGTCCAA	98aa; *Xba*I, *Nco*I

PS6-25-F	GC tctaga ccatgg AAGCTTCTGATGTCCAGTC	274aa; *Xba*I, *Nco*I

PS6-26-R	CCG ctcgag ggtacc CTCAGAGCTTAGTTGCCAGAG	full-length reverse primer without stop codon; *Xho*I *Kpn*I

PS9-5-F	CTAG ccatgg *GA *ATGGCAGACCAAGAGCG	1aa; *Nco*I

PS9-6-R	CG ggatcc AACGTCCAATTTCAAGG	full-length reverse primer without stop codon; *Bam*HI

PS9-9-F	CG ggatcc ATGGCAGACC AAGAGCG	1aa; *Bam*HI

PS9-10-R	CCG ctcgag AACGTCCAATTTCAAGG	full-length reverse primer without stop codon; *Xho*I

PS9-11-F	CCG gaattc TCTCATCTCCCTAACC	76aa; *Eco*RI

PS9-12-R	CG ggatcc CAAATACATTAAAAAGCC	207aa; *Bam*HI

PS9-13-F	CCG gaattc GGTGAAAATCCAAACTC	208aa; *Eco*RI

PS9-14-R	CG ggatcc GTGATTAACTTCTTTATTTG	248aa; *Bam*HI

PS9-15-F	CCG ctcgag *CT *ATGGCAGACCAAGAGCG	1aa; *Xho*I

PS9-16-R	ggtacc ggatcc TCAAACGT CCAATTTCAAG	full-length reverse primer, *Kpn*I, *Bam*HI

PS9-17-F	gaattc gtcgac ATGGCAGACCAAGAGC	1aa, *Eco*RI, *Sal*I

PS9-18-F	gaattc gtcgac ATGTCGTTGTTGCCAAT	167aa, *Eco*RI, *Sal*I

PS9-19-F	gaattc gtcgac ATGTATATAAAAGGCTT	198aa, *Eco*RI, *Sal*I

^a ^Introduced restriction endonuclease sites are in lower case. Two extra nucleotides (italicized) were added to allow in-frame expression of fusion proteins of interest.

^b ^Numbered according to P6 amino acid sequence. The F or R designation in the primer names denotes whether the primer is a forward (5′) or reverse (3′) primer, respectively.

To generate yeast plasmids for the two-hybrid assay, P6 ORF was amplified using primers PS6-4/PS6-5. PCR products were digested with *Nco*I/*Bam*HI, and then ligated into the same sites of pGADT7 to generate pGADT7-S6. Vectors pGADT7-S9-1 and pGBKT7-S9-1 were created using similar strategies. P6 ORF was amplified using primers PS6-6/PS6-9 and the PCR products were ligated to pMD19-T. The clone was digested with *Bam*HI/*Sal*I, and P6 *Bam*HI/*Sal*I-fragments were ligated into the corresponding sites of pGBKT7 to obtain pGBKT7-S6. P6 *Nco*I/*Bam*HI-fragments excised from the pMD19-T intermediates (containing P6^274-703^, P6^395-703^, P6^395-659^) were ligated into *Nco*I/*Bam*HI sites of pGADT7 and pGBKT7 to generate vectors expressing truncations fused with AD or BD. pGADT7-S6 was digested with *Eco*RI/*Bam*HI and *Eco*RV, and the liberated fragments were ligated into pGADT7 *Eco*RI/*Bam*HI and *Sma*I to obtain constructs expressing AD-P6^341-792 ^and AD-P6^271-703^. P6 *Bam*HI/*Xho*I-fragment from the intermediate harbouring P6^1-449 ^was inserted into the corresponding sites of pET30a, and then the clone was digested with *Nco*I/*Xho*I. The liberated fragment was cloned into the *Nco*I/*Xho*I-digested pGADT7 to obtain constructs expressing AD-P6^1-449^. P6 *Nco*I/*Xho*I-fragments excised from the pMD19-T intermediates (containing P6^98-792 ^and P6^274-792^) were ligated into the same sites of pGBKT7 to generate vectors expressing truncations BD-P6^98-792 ^and BD-P6^274-792^. Similar strategies were used to generate the constructs expressing P9-1 mutants fused with BD.

To obtain construction binary vectors for BiFC, the intermediate which contained P6^274-703 ^was digested with *Xba*I/*Bam*HI, and the P6^274-703 ^fragment was then inserted into the same sites of pSPYNE-35S and pSPYCE-35S to generate vectors expressing P6^274-703^-NE and P6^274-703^-CE.

The primers used in the experiments are shown in Table 1. All clones derived from the PCR products were verified by sequencing, and the recombinant plasmids were confirmed by restriction analyses.

### YTH and *β*-galactosidase assays

Yeast transformations were conducted using the small-scale lithium acetate method. Two-hybrid assays were performed using the Matchmaker GAL4 Two-Hybrid System3 (Clontech), according to the manufacturer's protocols. Cotransformants were plated on synthetic defined (SD) minimal medium minus adenine, histidine, leucine, and tryptophan (SD/-Ade/-His/-Leu/-Trp), and positive yeast colonies that could grow on the auxotrophic medium were lysed in liquid nitrogen and then tested for *β*-galactosidase activity as mentioned in the *β*-galactosidase colony-lift filter assay.

### Transient expression of protein in onion cells

To introduce plasmid DNA into onion epidermal cells, particle bombardment was conducted using a helium-driven particle accelerator PDS-1000/He (Bio-Rad). 2-5 μg plasmid DNA in 5 μL distilled water were mixed with 8 μL of a 60 mg/mL 1.0-μm-diameter gold particle solution, 20 μL of 2.5 M CaCl_2_, and 8 μL of 0.1 M fresh prepared spermidine. The resultant suspension was incubated for 10 min with intermittent mixing every 1 to 2 min at room temperature. The golden particles coated with plasmid DNA were collected by 5-s pulse centrifugation. After the supernatant was removed, the pellet was washed with 100 μL of 70% cold ethanol followed by the same volume of 100% cold ethanol, and then suspended in 10 μL 100% ethanol. After being dried on the center of an aluminum foil rupture disk, the gold particles were bombarded into onion cells under a vacuum of 28 mm Hg with 6-cm target distances. The bombarded onion epidermal cells were cultured on 0.6% agar with 2,4-D-free MS medium at 25 °C in darkness. Fluorescence signals were detected at 16 to 24 h after bombardment [37,38].

### Subcellular localization of RBSDV P6 derivatives and BiFC assay in *N. benthamiana *leaves

Different binary plasmids were transformed into *A. tumefaciens *EHA105 by a freeze-thaw method. Cultures of EHA105 harbouring a relevant binary plasmid were grown in LB medium containing rifampicin (50 g/ml) and kanamycin (100 g/ml) at 28 °C for 16 h. For expression of different fusions, EHA105 strains containing the pGDR derivatives and pGDp19 plasmid were resuspended and adjusted to an OD_600 _of 0.5:0.3 with infiltration medium (10 mM MES, pH 5.6, 10 mM MgCl_2_, 150 mM acetosyringone). For the BiFC assay, *Agrobacterium *cultures containing the BiFC plasmids and the pGDp19 plasmid were resuspended at a final OD_600 _of 0.5:0.5:0.3. The cells were incubated at room temperature for 2 to 4 h, and then infiltrated into 5-6-week-old *N. benthamiana *leaves. Underside epidermal cells of tobacco infiltrated leaves were assayed for fluorescence 48-96 h after infiltration [39].

### Laser-scanning confocal microscopy

Fluorescence analysis was performed using a Nikon ECLIPSE TE2000-E inverted fluorescence microscope equipped with a Nikon D-ECLIPSE C1 spectral confocal laser scanning system. GFP and YFP were both detected with an excitation at 488 nm and emission capture at 550-590 nm. DsRed2 was excited at 543 nm using a 543-nm helium neon laser, and the emission was captured at 570 to 600 nm [40]. For analysis of coexpression assays, multi-tracking was used to prevent emission cross-talk between the channels.

## Competing interests

The authors declare that they have no competing interests.

## Authors' contributions

QW carried out most of the experiments and wrote the manuscript. TT and WW anticipated the construction of the recombinants. YZ provided useful advice and anticipated in the protein transient expression assays. CH, DL and JY conceived of the study and participated in its design and coordination. All authors read and approved the final manuscript.

## Supplementary Material

Additional File 1 **Transient expression of P6 fused with GFP in *N. benthamiana *protoplasts**. Tobacco protoplasts were isolated and transfected using a modified PEG method. Punctata VLS of different sizes were prevalently formed in *N. benthamiana *protoplasts expressing P6-GFP, while diffuse GFP fluorescence was observed in the nucleus and cytoplasm of the cells expressing free GFP. The results were observed 16 h after PEG transfection. Bars, 20 μm. Click here for file

Additional File 2 **Transient expression of P6 truncations fused with DsRed2 in *N. benthamiana *protoplasts**. DsRed-P6^274-792^, DsRed-P6^395-703 ^and DsRed-P6^395-659 ^formed discrete bright aggregate-like structures in the *N. benthamiana *protoplasts, while a weak and diffuse fluorescence was also detected in the cytoplasm. Free DsRed2 resulted in a diffuse pattern of fluorescence that was both nuclear and cytoplasmic. Bars, 20 μm. Click here for file

Additional File 3 **Transient expression of DsRed-P9-1 and P9-1-GFP in *N. benthamiana *cells or protoplasts**. The plasmids expressing DsRed-P9-1 and P9-1-GFP were introduced into tobacco cells by agro-infiltration assay or PEG transfection, respectively. Both DsRed-P9-1 and P9-1-GFP resulted in a pattern of diffuse and uniform fluorescence distribution in the cytoplasm of *N. benthamiana *cells or protoplasts, which indicated that P9-1 is unable to form aggregate-like structures when expressed alone in tobacco cells. Bars, 20 μm. Click here for file
