# Supplementary figures and images for: Rice black-streaked dwarf virus P6 self-interacts to form punctate, viroplasm-like structures in the cytoplasm and recruits viroplasm-associated protein P9-1
Source: Virol J. 2011 Jan 18;8:24. doi: 10.1186/1743-422X-8-24 (PMC3032713; doi:10.1186/1743-422X-8-24)

## Slide 1
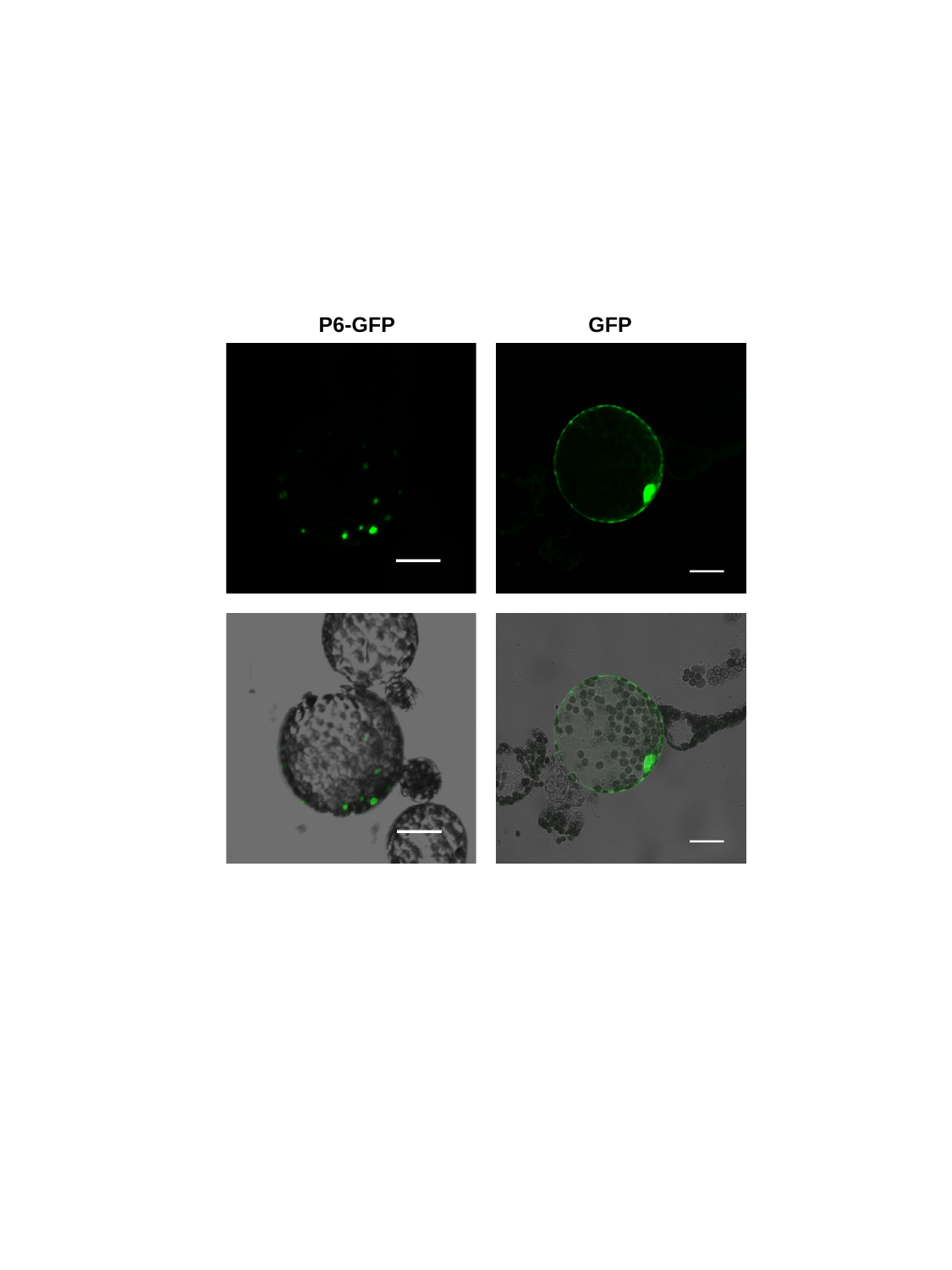

P6-GFP
GFP

Supplement: Additional File 1 — Transient expression of P6 fused with GFP in N. benthamiana protoplasts. Tobacco protoplasts were isolated and transfected using a modified PEG method. Punctata VLS of different sizes were prevalently formed in N. benthamiana protoplasts expressing P6-GFP, while diffuse GFP fluorescence was observed in the nucleus and cytoplasm of the cells expressing free GFP. The results were observed 16 h after PEG transfection. Bars, 20 μm. [file 1743-422X-8-24-S1.PPT]

## Slide 1
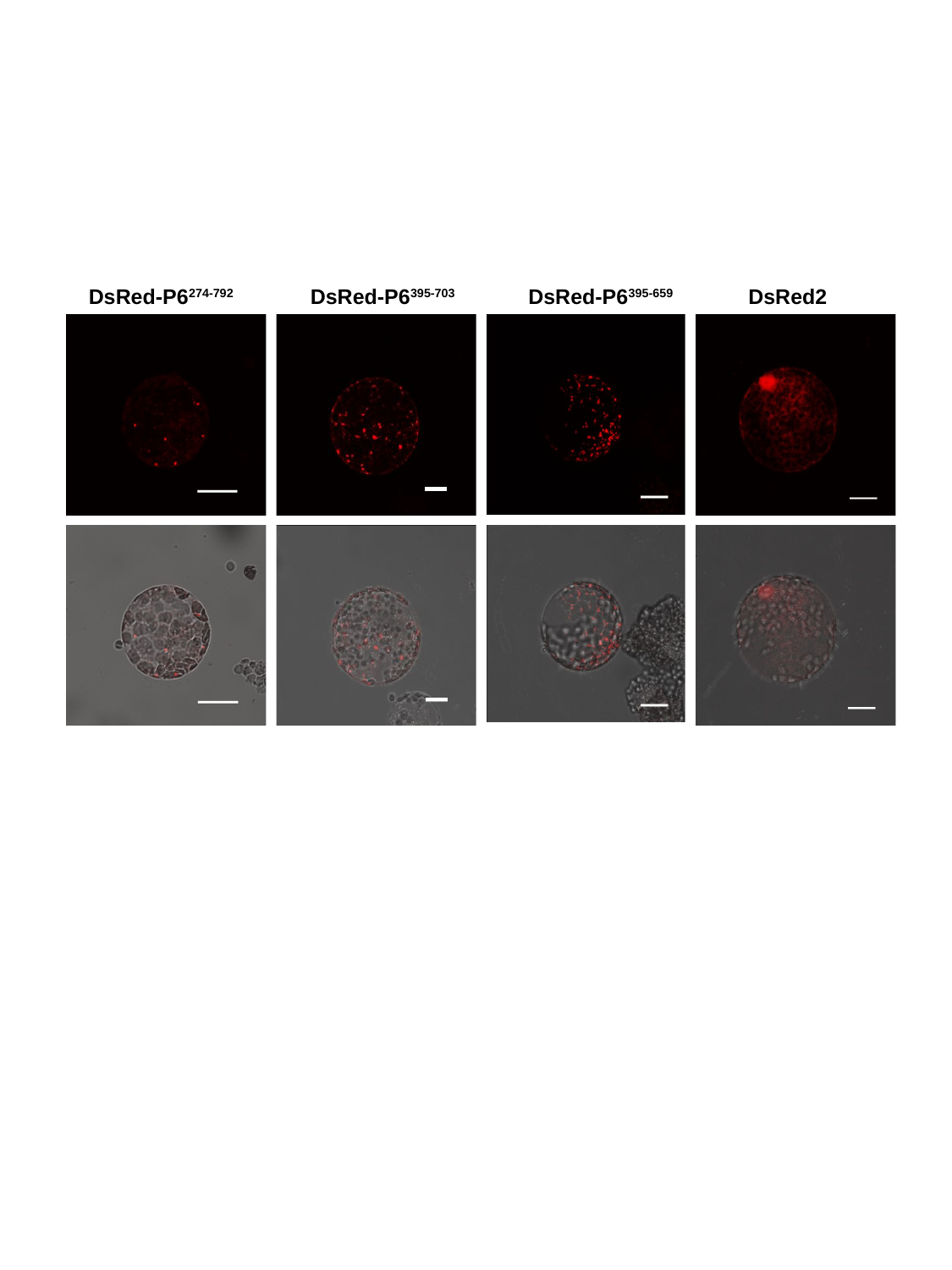

DsRed-P6274-792
DsRed-P6395-703
DsRed-P6395-659
DsRed2

Supplement: Additional File 2 — Transient expression of P6 truncations fused with DsRed2 in N. benthamiana protoplasts. DsRed-P6274-792, DsRed-P6395-703 and DsRed-P6395-659 formed discrete bright aggregate-like structures in the N. benthamiana protoplasts, while a weak and diffuse fluorescence was also detected in the cytoplasm. Free DsRed2 resulted in a diffuse pattern of fluorescence that was both nuclear and cytoplasmic. Bars, 20 μm. [file 1743-422X-8-24-S2.PPT]

## Slide 1
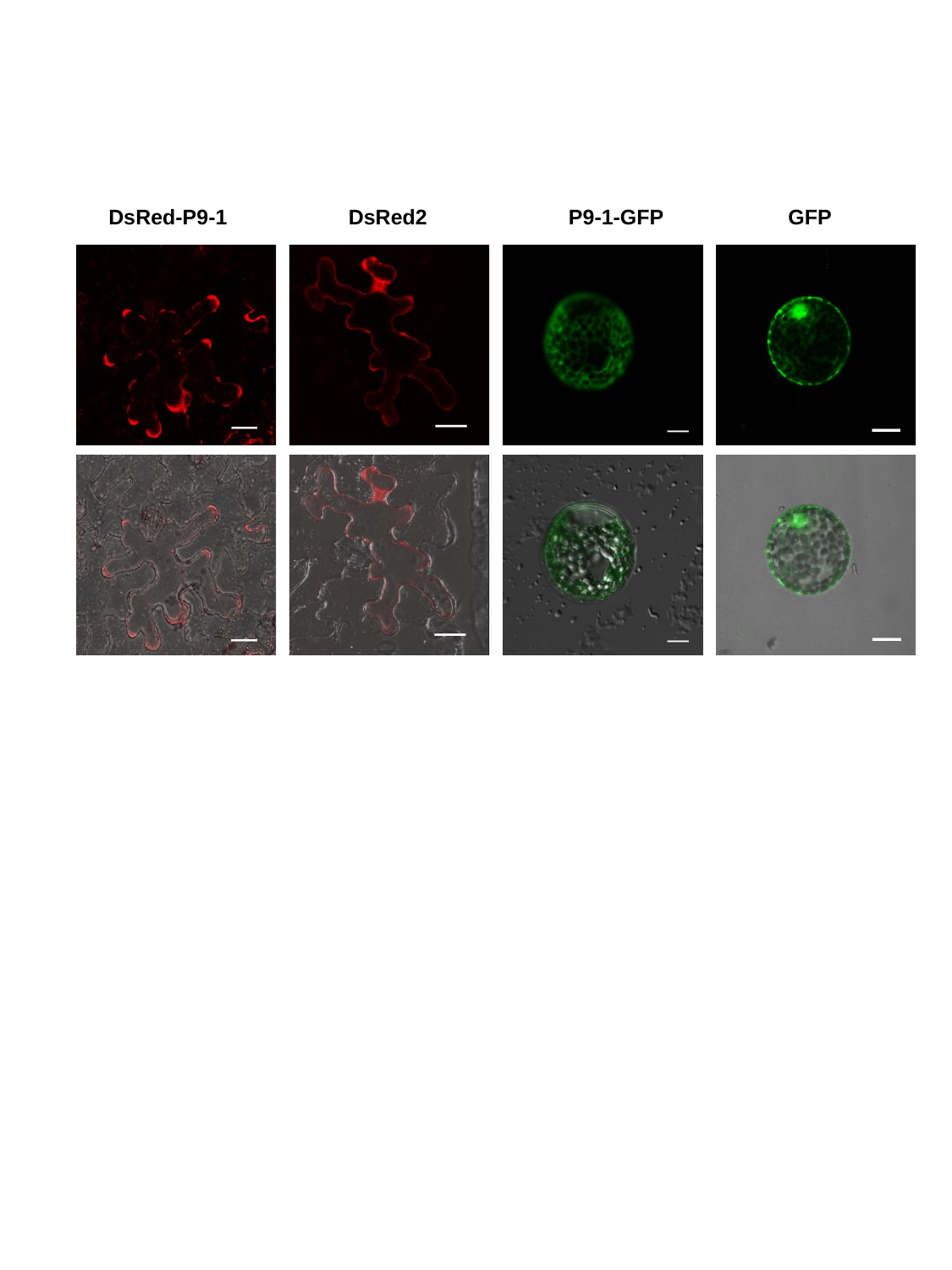

DsRed-P9-1
DsRed2
P9-1-GFP
GFP

Supplement: Additional File 3 — Transient expression of DsRed-P9-1 and P9-1-GFP in N. benthamiana cells or protoplasts. The plasmids expressing DsRed-P9-1 and P9-1-GFP were introduced into tobacco cells by agro-infiltration assay or PEG transfection, respectively. Both DsRed-P9-1 and P9-1-GFP resulted in a pattern of diffuse and uniform fluorescence distribution in the cytoplasm of N. benthamiana cells or protoplasts, which indicated that P9-1 is unable to form aggregate-like structures when expressed alone in tobacco cells. Bars, 20 μm. [file 1743-422X-8-24-S3.PPT]
